# Supplementary material for: Diagnostic Performance of Conventional X-ray for Detecting Foreign Bodies in the Upper Digestive Tract: A Systematic Review and Diagnostic Meta-Analysis
Source: Diagnostics (Basel). 2021 Apr 27;11(5):790. doi: 10.3390/diagnostics11050790 (PMC8145745; doi:10.3390/diagnostics11050790)
Supplement: Supplementary file 1 [file diagnostics-11-00790-s001.zip › diagnostics-1163394-supplementary.pdf]

Diagnostic performance of conventional X-ray for detecting foreign bodies in the upper digestive tract: A systematic review and diagnostic meta-analysis

Ta-Wei Yang[1,2], Yi-Chung Yu[3-4], Yen-Yue Lin[1,2], Shih-Chang Hsu[3-4], Karen Chia-Wen Chu[3-4], Chin-Wang Hsu[3-4], Chyi-Huey Bai[5], Cheng-Kuang Chang[6], Yuan-Pin Hsu[3-4,7*]

Supplementary Online Content

Table S1. Search Strategy
Table S2. Type and location of foreign bodies in the included studies
Table S3. Summary performance estimates of diagnostic parameters
Figure S1. Methodological quality of the included 17 studies
Figure S2. Deeks' funnel plot asymmetry test for publication bias
Figure S3. Sensitivity analysis after the exclusion of five studies with a sample size of <100

Table S1. Search strategy

Search	Query	Items found
Recent queries in PubMed on August 1, 2020		
Search	Query	Items found
1	neck	349,200
2	soft tissue	151,646
3	radiograph*	557,278
4	X-ray	1,831,042
5	plain film*	5735
6	Foreign	267,906
7	body or bodies	1,512,914
8	object*	2,500,622
9	impact*	1,108,626
10	obstruct*	348,057
11	ingest*	101,846
12	(((soft tissue) OR (radiograph*)) OR (X ray)) OR (plain film*)	2,081,644
13	(neck) AND ((((soft tissue) OR (radiograph*)) OR (X ray)) OR (plain film*))	74,309
14	((((body or bodies) OR (object*)) OR (impact*)) OR (obstruct*)) OR (ingest*)	4,977,452
15	(foreign) AND (((((body or bodies) OR (object*)) OR (impact*)) OR (obstruct*)) OR (ingest*))	89,547
16	((neck) AND ((((soft tissue) OR (radiograph*)) OR (X ray)) OR (plain film*))) AND ((foreign) AND (((((body or bodies) OR (object*)) OR (impact*)) OR (obstruct*)) OR (ingest*)))	1223
Recent queries in Embase on August 1, 2020		
Search	Query	Items found
1	“soft tissue”/exp OR “soft tissue” OR (soft AND (“tissue”/exp OR tissue))	210,462
2	radiograph*	771,399
3	plain AND film*	9706
4	“x ray”/exp OR “x ray” OR (x AND ray)	611,800
5	“neck”/exp OR neck	499,596
6	foreign	129,661
7	“body”/exp OR body	2,265,711
8	Bodies	171,446
9	object*	3,684,881
10	impact*	1,614,907
11	obstruct*	618,034
12	ingest*	132,375
13	#1 OR #2 OR #3 OR #4	1,464,050
14	#5 AND #13	61,225
15	#7 OR #8 OR #9 OR #10 OR #11 OR #12	7,332,532
16	#6 AND #15	79,053
17	#14 AND #16	1420
Recent queries in the Cochrane Library on August 1, 2020		
Search	Query	Items found
1	soft tissue	8017
2	radiograph*	23,125
3	X-ray	17,920

4	plain film*	569
5	Neck	26,173
6	body or bodies	145,481
7	object*	366,043
8	impact*	120,121
9	obstruct*	37,640
10	ingest*	16,668
11	Foreign	2797
12	#1 or #2 or #3 or #4	44,345
13	#5 and #12	2798
14	#6 or #7 or #8 or #9 or #10	563,726
15	#11 and #14	2326
16	#13 and #15	37
Recent queries in Web of Science on August 1, 2020		
Search	Query	Items found
1	soft tissue	119,675
2	radiograph*	171,111
3	X ray	1,202,558
4	plain film*	4754
5	Neck	216,838
6	body or bodies	1,445,916
7	object*	2,678,105
8	impact*	2,258,776
9	obstruct*	258,479
10	ingest*	100,400
11	Foreign	139,351
12	#4 OR #3 OR #2 OR #1	1,466,374
13	#12 AND #5	21,092
14	#10 OR #9 OR #8 OR #7 OR #6	6,073,427
15	#14 AND #11	45,510
16	#15 AND #13	245
Recent queries in Scopus on August 1, 2020		
Search	Query	Items found
1	TITLE-ABS-KEY (soft AND tissue)	214,307
2	TITLE-ABS-KEY (radiograph*)	834,148
3	TITLE-ABS-KEY (XAND ray)	2,157,877
4	TITLE-ABS-KEY (plain AND film*)	9623
5	TITLE-ABS-KEY (neck)	359,658
6	TITLE-ABS-KEY (body OR bodies)	2,946,863
7	TITLE-ABS-KEY (object*)	4,713,725
8	TITLE-ABS-KEY (impact*)	3,232,827
9	TITLE-ABS-KEY (obstruct*)	580,155
10	TITLE-ABS-KEY (ingest*)	150,610
11	TITLE-ABS-KEY (foreign)	357,009
12	(TITLE-ABS-KEY (soft AND tissue)) OR (TITLE-ABS-KEY (radiograph*)) OR (TITLE-ABS-KEY (x AND ray)) OR (TITLE-ABS-KEY (plain AND film*))	2,962,840
13	((TITLE-ABS-KEY (soft AND tissue)) OR (TITLE-ABS-KEY (radiograph*)) OR (TITLE-ABS-	60,849

	KEY (x AND ray)) OR (TITLE-ABS-KEY (plain AND film*))) AND (TITLE-ABS-KEY (neck))	
14	TITLE-ABS-KEY (#6 OR #7 OR #8 OR #9 OR #10)	12,380,247
15	(TITLE-ABS-KEY (#6 OR #7 OR #8 OR #9 OR #10)) AND (TITLE-ABS-KEY (foreign))	41,038
16	(((TITLE-ABS-KEY (soft AND tissue)) OR (TITLE-ABS-KEY (radiograph*)) OR (TITLE-ABS-KEY (x AND ray)) OR (TITLE-ABS-KEY (plain AND film*))) AND (TITLE-ABS-KEY (neck))) AND ((TITLE-ABS-KEY (#6 OR #7 OR #8 OR #9 OR #10)) AND (TITLE-ABS-KEY (foreign)))	305

* and # are symbols of the Boolean search

Table S2. Type and location of foreign bodies in the included studies

Study	Type of FB, N (%)	Location of FB, N (%)
Luo et al., 2020	Fish bone, 12 (12.6%); a bony structure with or without meat, 58 (61.1%); food without bones, 13 (13.7%); drug tablet, 7 (7.4%); denture, 4 (4.2%); plastic material, 1 (1.1%)	N/A
Pitts-Tucker et al., 2019	Fish bone, 31 (100%)	Palatine tonsil, 11 (23.9%); tongue base, 11 (23.9%); vallecula, 10 (21.7%); pharyngeal wall, 6 (13.0%); piriform fossa, 3 (6.5%); esophagus, 3 (6.5%); uvula 1, (2.2%); epiglottis 1, (2.2%)
Malik et al., 2018	Foreign bodies, 240 (100%)	N/A
Kumar et al., 2018	Fish bone, 28 (100%)	Oral cavity, 3 (11.1%); oropharynx, 10 (37.0%); laryngopharynx, 11 (40.7%); esophagus, 3 (11.1%)
Yao et al., 2015	Fish bone, 84 (37.2%); food bolus, 80 (35.4%); chicken bones, 10 (4.4%); coins, 2 (0.9%); toothpick, 5 (2.2%); medicine tinfoil pack, 4 (1.8%); denture, 19 (8.4%); battery, 1 (0.4%); others, 21 (9.2%)	Oropharynx, 14 (8.3%); upper esophagus, 75 (44.6%); middle esophagus, 34 (20.2%); lower esophagus, 18 (10.7%); stomach, 21 (12.5%); duodenum, 2 (1.2%); anastomosis, 4 (2.4%)
Shih et al., 2015	Fish bone, 41 (49.4%); chicken bone, 20 (24.1%); pork bone, 12 (14.5%); food bolus, 4 (4.8%); pill with wrapper, 3 (3.6%); tooth, 1 (1.2%); tablet, 1 (1.2%); coin, 1 (1.2%)	Cervical esophagus, 45 (54.2%); intrathoracic esophagus, 29 (34.9%); abdominal esophagus, 9 (10.8%)
Park et al., 2013	Fish bone, 45 (100%)	Upper esophagus, 22 (47.8%); pharyngoesophageal junction, 10 (21.7%); trans junctional position, 7 (15.2%); hypopharynx, 5 (10.9%); oropharynx, 2 (4.3%)
Wu et al., 2008	Fish bone, 52 (76.5%); chicken bone, 6 (8.8%); duck bone, 4 (5.9%); goose bone, 2 (2.9%); pork bone, 1 (1.5%); drugs, 2 (2.9%); fish ball, 1 (1.5%)	Oropharynx, 11 (16.2%); larynx, 1 (1.5%); hypopharynx or cervical esophagus, 51 (75%); thoracic esophagus, 5 (7.4%)
Saki et al., 2008	Fish bone, 58 (9.3%); chicken bone, 222 (35.6%); beef bone, 152 (24.4%); meat, 68 (10.9%); foreign body, 52 (8.3%); denture, 48 (7.7%); glass, 23 (3.7%)	Cervical esophagus just below the cricopharyngeal muscle, 522 (84.1%); piriform sinus, 62 (9.9%); middle third of the esophagus, 24 (3.9%); distal third of the esophagus, 13 (2.1%)
Prakash et al., 2008	Meat bone, 103 (44.8%); coin, 80 (34.8%); meat bolus, 14 (6.1%); metallic foreign body, 13 (5.6%); vegetative foreign body, 6 (2.6%); battery, 5 (2.2%); denture, 5 (2.2%); electric foreign body, 4 (1.7%)	N/A
Akazawa et al., 2004	Fish bone, 31 (100%)	N/A

Lai et al., 2003	Fish bone, 336 (84.0%); chicken bone, 38 (9.5%); pork bone, 14 (3.5%); duck bone, 4 (1.0%); denture, 4 (1.0%); coin, 3 (0.7%); food bolus, 2 (0.5%)	Valleculae, 126 (31.4%); oropharynx, 69 (17.2%); pyriform fossa, 66 (16.5%); cricopharyngeal, 59 (14.7%); upper esophagus, 51 (12.7%); epiglottic fold, 30 (7.5%)
Wai Pak et al., 2001	Fish bone, 104 (90.4%); chicken bone, 2 (1.7%); coins, 8 (7.0%); glass pieces, 1 (0.9%)	Tonsils, 83 (72.2%); tongue base, 15 (13%); pharyngeal wall, 4 (3.4%); cricopharyngeal, 8 (6.8%); uvula, 1 (0.9%); unknown/dislodged, 4 (3.4%)
Ciriza et al., 2000	Fish bone, 19 (29.7%); other bones, 16 (25%); food, 22 (34.4%); coin, 4 (6.3%); shell, 1 (1.6%); medication tablet, 1 (1.6%); clam, 1 (1.6%)	Hypopharynx, 4 (6.3%); left piriform fossae, 2 (3.1%); right piriform fossae, 2 (3.1%); cricopharyngeal area, 12 (18.8%); cervical esophagus, 24 (37.5%); middle esophagus, 7 (10.9%); distal esophagus, 9 (14.1%); stomach, 3 (3.1%); duodenum, 1 (1.6%)
Eliashar et al., 1999	Fish or chicken bone, 30 (100%)	N/A
Evans et al., 1992	Fish bone, 100 (100%)	Valleculae, 34 (34%); posterior third of the tongue, 29 (29%); tonsil, 23 (23%); oropharyngeal wall, 4 (4%); piriform fossa, 3 (3%); cricopharyngeal, 3 (3%); cervical esophagus, 2 (2%); thoracic esophagus, 1 (1%); larynx, 1 (1%)
Ngan et al., 1989	Fish bone, 117 (100%)	Oral cavity, 6 (5.1%); oropharynx, 73 (62.4%); laryngopharynx, 18 (15.4%); hypopharynx, 18 (15.4%); esophagus, 2 (1.7%)

FB, foreign body; NA, not available

Table S3. Summary of the performance estimates of diagnostic parameters

Parameter	Estimate	95% CI
Sensitivity	0.58	0.36–0.77
Specificity	0.94	0.87–1.98
PLR	10.1	4.4–23.3
NLR	0.44	0.27–0.74
DOR	23	7–70

CI, confidence interval; PLR, positive likelihood ratio; NLR, negative likelihood ratio; DOR, diagnostic odds ratio

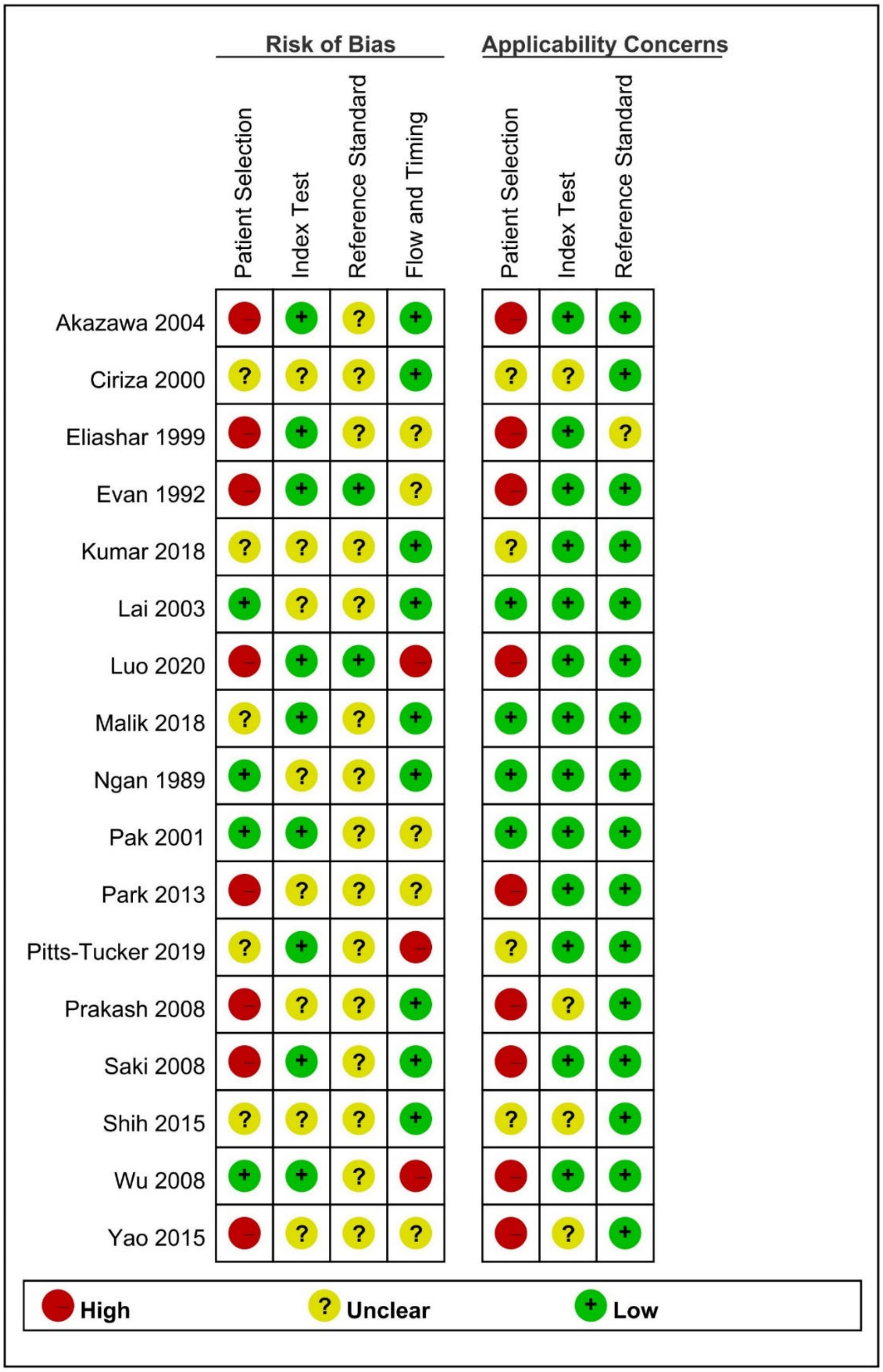

Figure S1. Methodological quality of included 17 studies

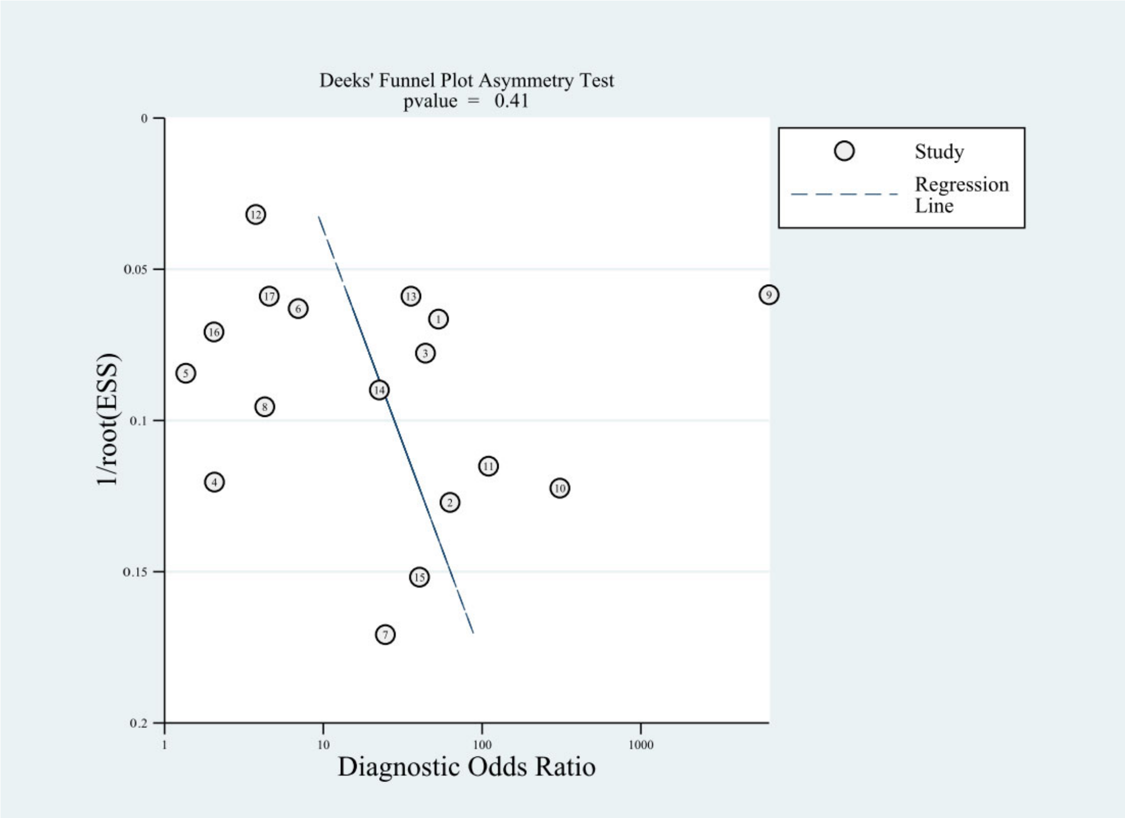

Figure S2. Deeks’ funnel plot asymmetry test for publication bias

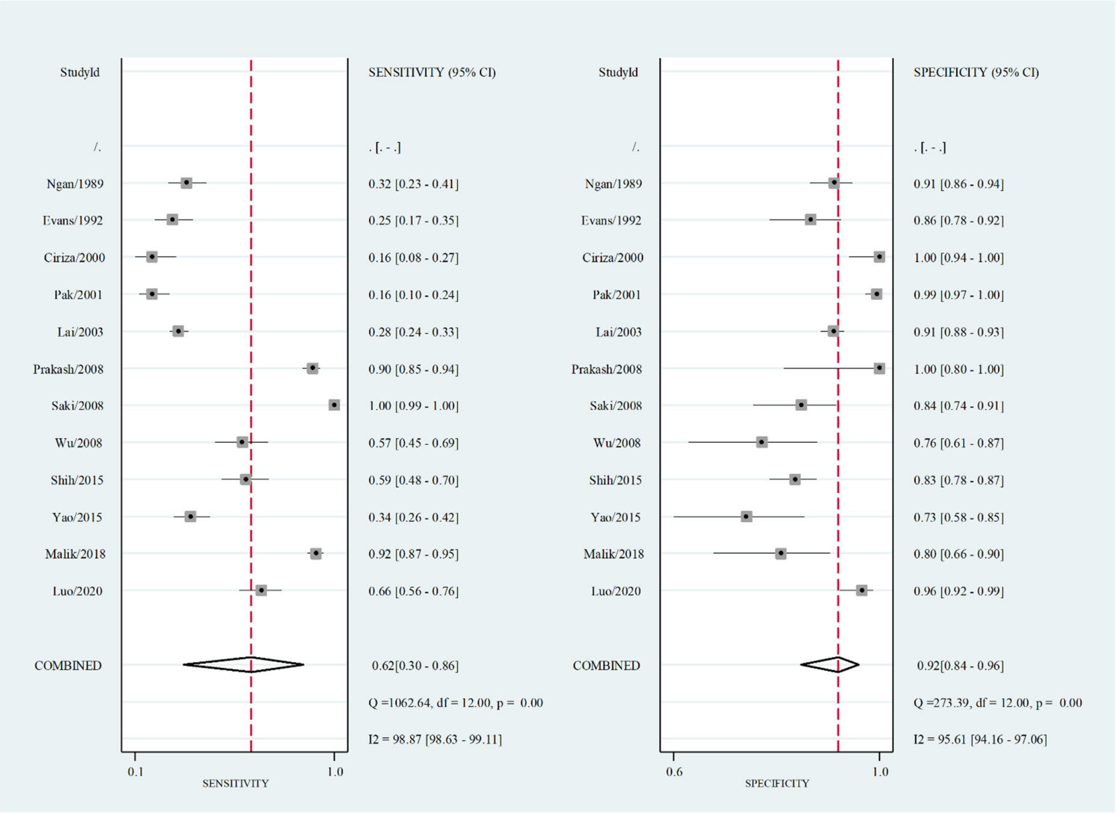

Figure S3. Sensitivity analysis after the exclusion of five studies with a sample size of <100
